# Amino acid supplements and metabolic health: a potential interplay between intestinal microbiota and systems control

**DOI:** 10.1186/s12263-017-0582-2

**Published:** 2017-10-04

**Authors:** Francesco Bifari, Chiara Ruocco, Ilaria Decimo, Guido Fumagalli, Alessandra Valerio, Enzo Nisoli

**Affiliations:** 1 Laboratory of Cell Metabolism and Regenerative Medicine, Department of Medical Biotechnology and Translational Medicine, University of Milan, Milan, Italy; 2 Center for Study and Research on Obesity, Department of Medical Biotechnology and Translational Medicine, University of Milan, 20129 Milan, Italy; 3 Section of Pharmacology, Department of Diagnostics and Public Health, University of Verona, Verona, Italy; 4 Department of Molecular and Translational Medicine, University of Brescia, Brescia, Italy

**Keywords:** Aging, Branched-chain amino acids, Diabetes, Essential amino acids, Gut microbiota, Obesity, Healthspan, Microbes, Short-chain fatty acids, Supplement

## Abstract

Dietary supplementation of essential amino acids (EAAs) has been shown to promote healthspan. EAAs regulate, in fact, glucose and lipid metabolism and energy balance, increase mitochondrial biogenesis, and maintain immune homeostasis. Basic science and epidemiological results indicate that dietary macronutrient composition affects healthspan through multiple and integrated mechanisms, and their effects are closely related to the metabolic status to which they act. In particular, EAA supplementation can trigger different and even opposite effects depending on the catabolic and anabolic states of the organisms. Among others, gut-associated microbial communities (referred to as gut microbiota) emerged as a major regulator of the host metabolism. Diet and host health influence gut microbiota, and composition of gut microbiota, in turn, controls many aspects of host health, including nutrient metabolism, resistance to infection, and immune signals. Altered communication between the innate immune system and the gut microbiota might contribute to complex diseases. Furthermore, gut microbiota and its impact to host health change largely during different life phases such as lactation, weaning, and aging. Here we will review the accumulating body of knowledge on the impact of dietary EAA supplementation on the host metabolic health and healthspan from a holistic perspective. Moreover, we will focus on the current efforts to establish causal relationships among dietary EAAs, gut microbiota, and health during human development.

## Background

Dietary supplementation with essential (EAAs) and/or branched-chain amino acids (BCAAs) regulates metabolism and energy balance by directly affecting peripheral tissues, such as muscles, adipose tissue, and liver [1]. Moreover, EAA supplementation promotes cardiac and skeletal muscle mitochondrial biogenesis [2–4], prevents oxidative damage [5], enhances muscle protein synthesis and physical endurance [2, 6–9], reduces body weight [10–13], and increases immune function [14, 15]. Altogether, these effects have been shown to improve the healthspan and metabolic health [16]. Notably, the effect of EAAs drastically changes when they act in catabolic or anabolic conditions [1]. In catabolic states, EAAs represent mostly energy substrates, while in anabolic conditions EAAs fuel protein synthesis and cell growth. Recently, microbial communities present in the gastrointestinal tract, collectively termed the gut microbiota, have emerged as important regulators of metabolism [17–29] and immune homeostasis [30–41]. The human gut is associated with a diverse microbial community that is composed mainly of bacteria [19], but also includes methanogenic archaea (mainly *Methanobrevibacter smithii*), viruses (mainly phage), fungi, yeasts, and protozoa [42–45]. Metagenomic sequencing showed that bacterial communities usually consist of hundreds or thousands of bacterial taxa, principally pertaining to two phyla: *Firmicutes* and *Bacteroidetes* [19]. This ensemble of organisms has co-evolved with the human host [46] and extends the coding potential of human genome with 500-fold more genes [44, 47]. It has an essential role in altering the absorption, metabolite transformations, and energy storage [17, 23, 25, 48].

**Fig. 1:**
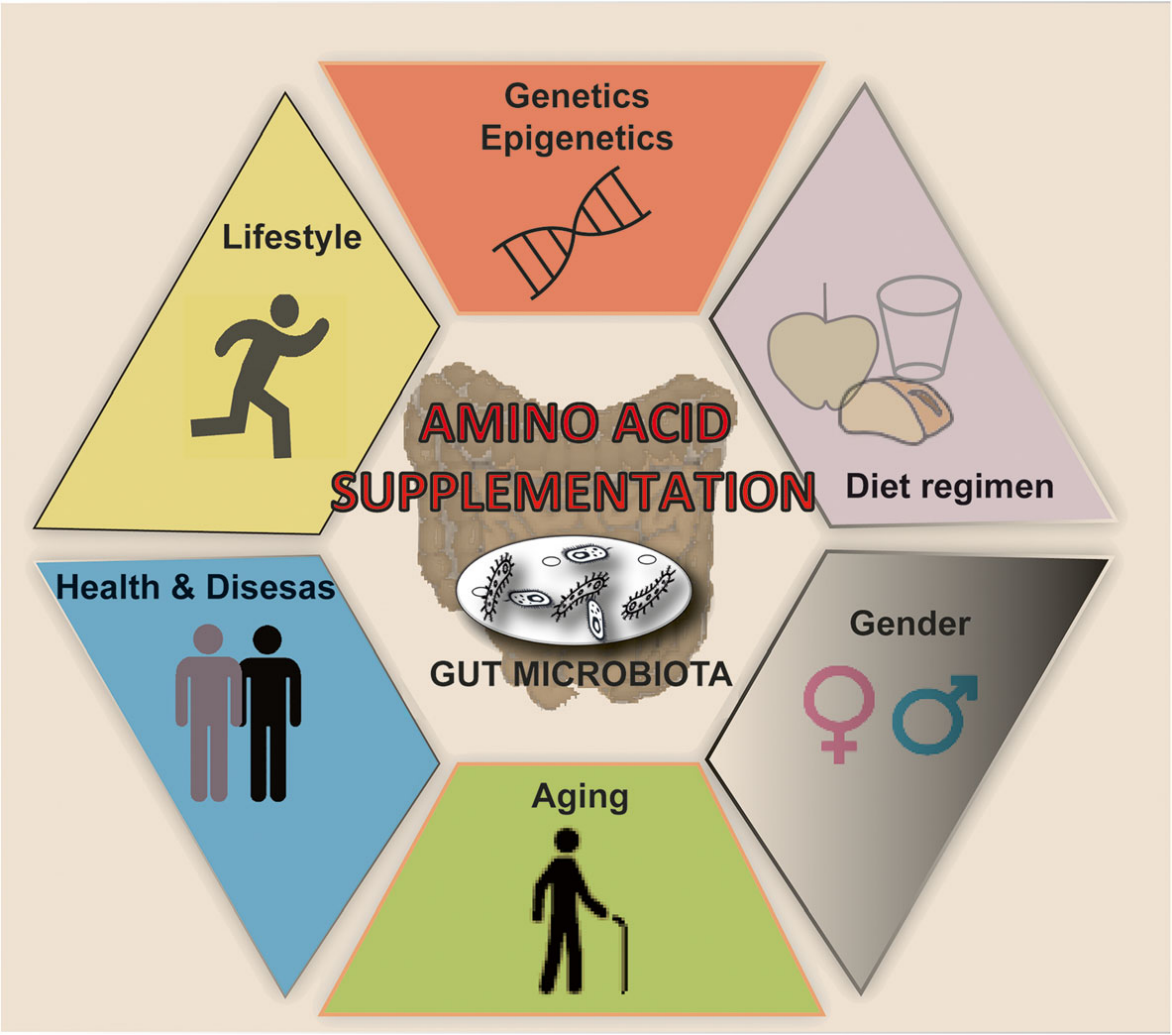
A large panel of factors can modulate the effects of specific amino acid supplements on gut microbiota. Gut microbiota owns a characteristic plasticity, and a lot of factors can modulate its composition, including genetic, epigenetic, and environmental factors (e.g., diet regimen and lifestyle), as well as aging, gender, and healthy or pathological conditions. Dietary supplementations with peculiar amino acid mixtures take place in this complex panorama

Comparing germ-free mice with otherwise syngeneic and conventionally raised mice allows understanding that the gut microbiota influences concentrations of the most metabolites detected in plasma [28]. Several of these circulating metabolites, such as bile acids and short-chain fatty acids, regulate function and homeostasis of diverse organs and tissues in a system-controlled manner. Gut microbiota can rapidly respond to large changes in diet [49–57], potentially facilitating the diversity of human dietary lifestyles and contributing to the host metabolic phenotype. Dietary EAAs have been suggested to modulate the intestinal immune system, in addition to their roles as building blocks for protein synthesis, nutrient signals, and modulators of gene expression [58–60]. Furthermore, a BCAA-enriched mixture (BCAAem) has been shown to rejuvenate the age-related modifications of gut microbiota [60]. In this review we will summarize the effect of dietary EAA supplements, highlighting the potential interactions between EAAs and gut microbiota (Fig. 1).

### EAA supplementation affects metabolism and health

In conditions of dietary nitrogen balance, the adult protein turnover is approximately 250 g/day [61]. Whole body protein synthesis in humans drastically decreases with age being 10 times less in elderly compared to newborns. Similarly, the protein catabolism also decreases with age. These parameters can largely change in conditions of nutrient deprivation and in disease states, for example, in traumatized or septic subjects [62]. In healthy gut, dietary EAAs are efficiently taken up by different amino-acid transporters in the enterocytes of proximal jejunum [63]. Moreover, EAAs, in particular leucine, have been shown to act as potent nutrient signals. At the molecular level, it has been shown that intracellular leucine concentration can be sensed by the multiprotein complex leucyl-tRNA synthetase [64, 65], which activates the mechanistic target of rapamycin (mTOR) kinase. Amino acid-induced mTOR activation regulates protein, lipid, and nucleotide synthesis, as well as inhibits autophagy.

Dietary BCAAem supplementation has been shown to improve motor performance and physical endurance [2]. In adult mice, mTOR signaling activated by BCAAem enhances the mitochondrial biogenesis partly through increasing nitric oxide production [2]. In skeletal muscles of aged rats, BCAAem recovers the reduced basal and post-insulin mTOR and p70S6K activation and the impaired post-insulin Akt activation [66], and improves the age-associated loss of function and muscle mass [67]. BCAAem has been reported also to increase de novo synthesis of proteins and to reduce the protein breakdown, with rescue of rosuvastatin-induced myopathy [5].

**Fig. 2:**
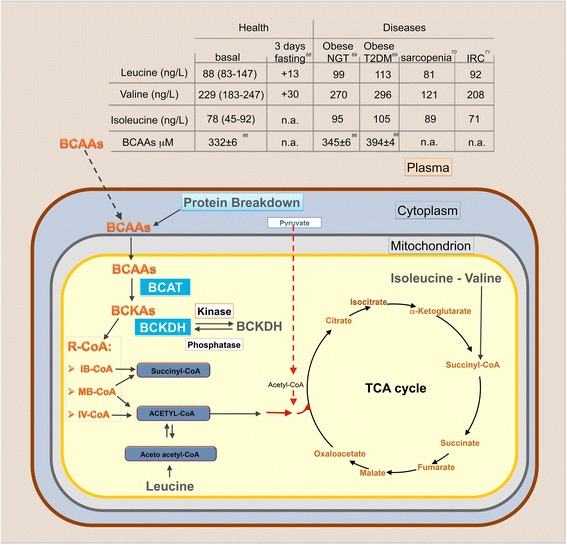
Biochemistry of BCAAs. Plasma (brown), cytosolic (light blue) and mitochondrial (gray) compartments are depicted. Concentrations of branched-chain amino acids (BCAAs) in physiological and pathological conditions are reported in the table. BCAAs can both enter the cell from the plasma and be produced by protein breakdown. Intracellular BCAAs are transaminated in mitochondria by branched-chain aminotransferase (BCAT). The resulting branched-chain α-keto acids (BCKAs, especially α-keto acid from leucine) inhibit branched-chain α-keto acid dehydrogenase kinase, resulting in elevation of the active state of the rate limiting enzyme branched-chain α-keto acid dehydrogenase complex (BCKDH). BCAAs can be oxidized to generate ATP. Carbon originating from BCAAs enters the tricarboxylic acid (TCA) cycle as acetyl-CoA for complete disposal as CO_2_. Isoleucine and valine provide carbon for anaplerotic conversion of propionyl-CoA to succinyl-CoA. IB-CoA, isobutyryl-coenzyme A; IV-CoA, isovaleryl-coenzyme A; MB-CoA, α-methylbutyryl-coenzyme A; R-CoA, acyl-coenzyme A

Circulating EAA concentrations are influenced by fasting and pathological conditions [68–71] (Fig. 2). During starvation, EAA metabolism is directed toward oxidation to generate ATP. This process is regulated by activation of AMP-activated kinase (AMPK), a master sensor of the energy balance [72, 73]. BCAA supplementation has been successfully tested in acute and severe catabolic conditions, including burns and trauma [62]. In dialysis patients, the correction of the plasma amino-acid profile, through administration of EAAs, reduces proteinuria and delays the progression of renal disease [74–76]. Moreover, the BCAA supplementation improves prognosis and quality of life in patients with liver cirrhosis [77, 78].

Different catabolic states, including starvation and malnutrition, are known to impair immune homeostasis. In particular, the dietary restriction of amino acids impairs cytotoxic T lymphocytes and natural killer cell function [79], in addition to reduce lymphocyte proliferation [14]. In elderly people, protein malnutrition is one of the major causes of immune dysfunction [80]. Interestingly, dietary supplementation of BCAAs has been reported to reduce the incidence of infections acquired in geriatric long-term rehabilitation centers [15] as well as the risk of bacterial and viral infection in patients with decompensated cirrhosis [81, 82]. Furthermore, BCAAem supplementation may correct the nephropathy-linked anemia in hemodialysis patients fed low protein diet [83], as well as BCAAs ameliorate the post-intense exercise immunosuppression [14]. In obesity, insulin resistance, and type 2 diabetes mellitus (T2DM), the results of diverse and opposing anabolic and catabolic signals impair amino acid catabolism leading to the BCAA accumulation. Low circulating levels of adiponectin decrease BCAA catabolism through AMPK signal [84]. Resistin and visfatin, adipokines highly expressed in visceral fat, induce amino acid uptake and protein synthesis. EAAs have been reported to induce mTOR activation and increase insulin receptor substrate-1 (IRS-1) phosphorylation, thereby contributing to the development of impairment of insulin signaling [85]. Indeed, elevated blood BCAA levels have been found in conditions associated with insulin resistance, such as obesity and TDM2 [69, 86–88] (Fig. 2). Moreover, in TDM2 muscles, the BCAA metabolite 3-hydroxyisobutyrate increases endothelial fatty acid transportation, thus worsening the muscle insulin resistance [89]. On the other hand, in selected subsets of obese subjects, BCAA intake is associated with reduced body weight and body fat [90, 91]. Although the BCAAs have been shown to worsen TDM2 in obese subject, in a long-term randomized study of elderly people with T2DM [92], as well as in patients with chronic viral liver disease [93], BCAA supplementation improved metabolic control and ameliorated insulin resistance. BCAAem-supplemented middle-aged (16 months) mice showed increased expression of peroxisome proliferator-activated receptor γ coactivator-1 α (PGC-1α) and sirtuin 1 (SIRT1) and enhanced mitochondrial biogenesis and function in cardiac and skeletal muscles [2]. Further, BCAAem has been found to improve sarcopenia, that is the age-associated loss of muscle mass and function, in old rats [66] and to prevent muscle atrophy in mice bearing a cachexia-inducing tumor [94]. In middle-aged mice, BCAAem preserved muscle fiber size, improved physical endurance and motor coordination [2], decreased protein breakdown and protected against dexamethasone-induced soleus muscle atrophy in rats [8]. When administered orally at the beginning of rat senescence, BCAAem formula has been shown to maintain the health of kidney in aged rats [95], by inducing eNOS and vascular endothelial growth factor expression in kidney, thus increasing vascularization and reducing renal fibrosis. The EAA supplementation can ameliorate myocardial dysfunction in diabetic rats [96]. Moreover, improved vascularization and increased collagen deposition, in addition to the fibroblast proliferation, seem also to be involved in the cutaneous wound healing obtained with topical application of BCAAs and other essential amino acids in aged rats [97].

### Gut microbiota affects metabolism and health

Substantial evidence has been accumulated that gut microbial communities influence feeding, energy homeostasis, endocrine systems, and brain function. The human microbiota produces in gut lumen essential vitamins, including vitamin K, vitamin B12, biotin, folate, thiamine, riboflavin, and pyridoxine, which are absorbed by the intestine [98, 99]. During the recent years, it has become clear that the influence of the microbiome on health may be even more profound. In particular, it was well established that gut microbiota can generate and indirectly influence the concentration of proteins, including hormones, neurotransmitters, and inflammatory molecules with systemic effects linked to the development of many diseases, such as obesity, T2DM, or atherosclerosis [100–106].

Of particular interest is the bacterial production of short chain fatty acids (SCFAs), e.g., propionate, butyrate, and acetate from polysaccharide degradation, which can be used from the host tissues as substrates for energy metabolism [24]. The abundance in the gut of organisms from *Lachnospiraceae* family, or the ratio of *Firmicutes* to *Bacteroides* are often associated with the production of SCFAs, and their signal to gut enteroendocrine cells is mediated by binding to G protein-coupled receptors, namely GPR41 and GPR43 [107, 108]. Microbiota-derived butyrate has been reported to regulate levels of glucagon-like peptide 1 (GLP-1), which is produced by enterocytes [109–111]. GLP-1 enhances the glucose-dependent insulin secretion of the pancreatic beta cells [112]. Butyrate has been reported to act as an anti-inflammatory molecule, both on circulating immune cells and enterocytes, thus regulating gut-barrier properties [113–115]. Propionate production seems to be particularly relevant in human health, because it promotes satiety, and prevents the hepatic lipogenesis lowering thus cholesterol production [116–118].

Studies on microbial community structure by 16S rRNA gene sequencing have shown that relatively better energy-harvesting bioreactors promote energy storage, increasing the predisposition to obesity [25, 48]. The high ratio of *Firmicutes* to *Bacteroides*, observed in gut microbiota from obese patients, influences degradation of polysaccharides to SCFAs, in particular increasing acetate and decreasing butyrate production [29]. Increasing blood levels of acetate correlate with insulin resistance development, and they increase production of the orexigenic peptide ghrelin in the stomach [119]. Lower butyrate levels are linked to low level inflammation, which in turn decreases insulin resistance [17, 21, 26].

Studies in humans also suggest a role for the gut microbiota in T2DM. In particular, when treatment-naïve patients with metabolic syndrome received intestinal transplantation either from lean donors or from their own feces, recipients of feces from lean donors have a higher abundance of butyrate-producing bacteria linked to improvement of insulin sensitivity [26].

The composition of the gut microbiota is not constant during the lifetime of the host and changes with age [120], owed to several reasons, including alterations in intestinal functions or inflammatory processes [121–126]. Importantly, aging is associated with a shift in the ratio of *Bacteroidetes* to *Firmicutes* species [125, 127]. Indeed, in people over 60 years the total number of facultative anaerobic microbes (i.e. *Firmicutes*) increases, while the proportion of *Bifidobacteria* decreases in comparison to young subjects. The age-related changes of the gut microbiota have been found especially important in pathophysiological processes of the age-related disorders, such as frailty [128], neurodegeneration [129], cognitive decline [130], T2DM [131], and cardiovascular diseases [132, 133].

Different environmental factors can influence gut microbiota composition. Recent study demonstrated that exposure of mice to cold was accompanied to a change in microbiota taxa and caused browning of white adipose tissue, with increase of insulin sensitivity and heat production, in addition to weight loss when compared to control mice. Transplantation of the cold-adapted microbiota from cold exposed mice was sufficient to promote browning of white adipose tissues and to enhance insulin sensitivity in warm recipient mice [134].

Also the diet regimen rapidly and efficiently modifies the relative abundance of specific bacterial taxa [23] and virus [135]. The relevance of this fast, diet-induced dynamics is demonstrated by the microbial changes that are observed over 1–2 days when subjects add dietary fibers to their diet, or consume either a high-fiber and low-fat diet or a low-fiber and high-fat diet for 10 days [49]. From an evolutionary perspective, these changes were selected to maximize energy harvested by food. Indeed, microbiota acts in the intestine as a bioreactor, which permits degradation of otherwise indigestible dietary fibers (i.e., polysaccharides) [24]. Interpersonal variations in the virome are high, even in co-twins and their mothers sharing similar fecal bacterial communities [45]. Dietary intervention is associated with a change in the virome community to a new state, in which individuals on the same diet converged [135]. The functional relevance of this gut virome modification in metabolic health is, however, still unknown.

Modifications of the gut microbial composition affect host metabolism. Colonization of adult germ-free mice with a distal gut microbial community harvested from conventionally raised healthy mice causes a dramatic increase in body fat within 10–14 days, despite an associated decrease in food consumption [25]. Compared with microbiota of lean persons, intestinal microbial composition of obese individuals has less diversity [136], and it is characterized by lower prevalence of *Bacteroidetes* and a higher prevalence of *Firmicutes* [137]. Modification of gut microbiota, by either cohousing [138, 139] or antibiotic treatments [140] or transplantation of fecal microbiota from obese versus lean subjects, can modify obesity and metabolic phenotype [25, 27, 141]. These results reveal that transmissible and modifiable interactions between diet and microbiota influence host biology.

Likewise, gut microbiota composition is in turn influenced by a wide range of pathologies (e.g., asthma, arthritis, autism, obesity) [20, 142], and the disease phenotype can be transferred by microbiota transplantation. In fact, recent studies suggest that the microbiome may be a reflection of obesity (or leanness), as well as a cause of it. When obese people are maintained to reduced energy intake with diet and lose weight, the proportion of *Bacteroidetes* increases relative to *Firmicutes*. Conversely, when obese people resume their previous food consumption and gain weight, the proportion of *Firmicutes* increases [100].

In addition to promoting the absorption of monosaccharides from the gut lumen, the microbiota from obese mice selectively suppresses the production of the circulating lipoprotein lipase inhibitor Fiaf (fasting-induced adipose factor/angiopoietin-like protein 4/peroxisome proliferator-activated receptor γ angiopoietin-related protein), thus inducing de novo hepatic lipogenesis and deposition of triglycerides in adipocytes and liver [143]. Specific gut bacterial taxa in obese humans and animals metabolize faster phosphatidylcholine to choline, trimethylamine N-oxide (TMAO), and betaine taken with diet. TMAO has been shown to accelerate atherosclerosis by forward cholesterol transport via upregulation of macrophage scavenger receptors [144].

Interactions between the host immune system and gut microbiota prevent the overgrowth of otherwise under-represented or potentially harmful bacteria (for example, pathobionts) [30, 48]. On the other hand, gut microbiota itself shapes the development of the immune system through a vast range of signaling pathways [38]. Conventional or germ-free housing conditions impact peripheral immune system development in immunocompetent hosts [41].

Dietary fats increase the bile acid taurocholic, therefore altering gut microbiota and promoting colitis in genetically susceptible mouse model [145]. *Bacteroides*, and in particular *Bacteroides fragilis*, have been suggested to promote many immune functions of the host. The capsular polysaccharide A (PsA) of *Bacteroides fragilis* drives differentiation of interleukin-10 (IL-10)-secreting Treg cells. Monocolonization with *Bacteroides fragilis*, but not with a mutant lacking PsA, stimulates dendritic cell IL-12 production and corrects systemic T cell deficiencies and Th1/Th2 imbalance [145].

### Interaction between amino acid supplementation and gut microbiota

Given the link between gut microbiome and increasing risk to develop many diseases (e.g. obesity, T2DM, atherosclerosis), the manipulation of the gut microbiota might be a plausible strategy to reduce this risk [146]. Moreover, gut microbiota shows a great plasticity and it could be mostly modified by different factors, such as diets or supplements [53].

Dietary proteins and amino acids are important substrates for microbial fermentation in the colon [147], where they also serve as important nitrogen sources for the microbiota and support the growth of microbiota and host [51]. Several research groups have shown that maternal diet affects the colonization of the gut of pups [121], also through epigenetic mechanism [148]. Dietary amino acid intake increases the relative abundance of *Bacteroidetes* [27, 51]. In particular, supplementation with BCAAem to middle aged mice (15 months) caused a significant reduction in the *Firmicutes*/*Bacteroidetes* ratio [60]. Notably, this ratio was comparable to the ratio observed in the 11-month-old mice [60]. In line with these results, BCAAem supplementation significantly changed fructose, sucrose, and oleic acid gut metabolism. Much more information is needed about how the BCAAem supplementation modulates structural and functional properties of gut microbiota, and what is the link with the healthy effects of the BCAAem supplementation as previously described [1, 2].

Several common mechanisms are shared by healthy microbiota and dietary EAAs. Essential amino acids can increase the expression of intestinal β-defensin, the endogenous small cationic polypeptide that functions as a broad-spectrum antimicrobial substance, and thus potentially the amino acids greatly affect the gut microbial community composition [58, 59]. Furthermore, both EAAs and microbiota-derived SCFAs modulate the overall lipid balance and glucose metabolism [1, 18]. Similarly, oral administration of BCAAs or the microbiota-derived butyrate induce a dose-dependent increase in GLP-1 release from enterocyte [110, 149, 150], and decrease the expression of genes involved in the intestinal fatty acid transport and lipogenesis (i.e., acetyl-CoA carboxylase and fatty acid synthase). EAAs may also modify the abundance of gut metabolites by influencing cholecystokinin production and gallbladder contraction [151]. On the other hand, the intestinal dysbiosis alters gut barrier properties and, thus, it may reduce the diet-induced healthy effect [152].

Another point yet to be clarified is whether the supplementation of specific amino acid mixtures is able to modify metabolic diseases, including obesity and T2DM, via gut microbiota modifications, and how this effect can be permanent. The plasma concentration of some EAAs, including BCAAs, is higher in obese T2DM patients than healthy subjects [87]. Obese T2DM patients have also a peculiar gut microbiota composition [25]. In particular, the depletion of species from the *Bacteroides* genus in obese individuals is related to higher plasma concentration of BCAAs [153]. Of particular interest is the possibility that a subset of gut microbial communities directly synthetized EAAs by themselves, EAAs that would be subsequently absorbed by the intestinal mucosa. Many components of the gut microbiota possess the enzyme to directly synthetize essential amino acids [154, 155]. Indeed, the gut microbiota from obese subject synthetizes BCAAs, while it strongly decreases BCAA catabolism [153]. Thus, the plasma EAA concentrations may be not entirely the consequence of oral EAA intake. On the other hand, oral EAA administration may modify gut microbiota and, consequently, modify (i.e., reduce) paradoxically the plasma EAA concentrations.

Human body metabolism is the result of complex interactions between genetic, epigenetic, and environmental (primarily dietary and lifestyle) factors [156, 157]. Gut microbiota controls metabolism through physiologically important biochemical circuits, which are parts of energy consumption, storage, and distribution [124]. Gut microbiota plays key roles in controlling body metabolism, resistance to infections, and inflammation, as well as preventing autoimmunity disorders and cancer [18, 20, 38]. Brain-gut axis represents an important communication system that regulates whole body energy balance. Information exchange between gut and brain is essential for mammals to adapt to changing environments [38, 158]. EAA supplementation has been shown to improve the health span and metabolic health [16], by reducing body weight [159], increasing immune homeostasis [14, 15], promoting mitochondrial biogenesis [2–4], preventing oxidative damage [5], and enhancing muscle protein synthesis and physical endurance [2, 6–9].

Many aspects of amino acid effects on gut microbiota remain to be addressed, for example, whether the different effects of EAAs, acting either in catabolic or anabolic conditions, may be partially attributed to differences of the gut microbiota composition in these metabolic conditions. Moreover, whether EAAs through gut microbiota play some roles in human development, a number of hypotheses about microbial contributions to human development have been proposed in the past decade. One hypothesis is that maternal microbial ecology affects pregnancy, fetal development, and the future health of offspring [121]. Maternal vaginal, gut, and oral microbiota have relevant impact on fetal nutrition and development [121]. Alterations of maternal microbiota are thought to contribute to gestational adverse events, such as the preterm delivery. A compelling question is whether EAA supplements may favorably change the properties of the vaginal and gut microbes before, during, and after pregnancy. A recent study has shown that microbial community structure and function expand and diversify in all body sites from birth to age 4–6 weeks, and it then resembles microbiota from the corresponding maternal body site [160]. A related question is whether microbes associated with breast milk, which are highly personalized assemblages [161] and colonize the infant colon, such as some anaerobic species (*Bifidobacterium*), may be modified by maternal supplementation with EAAs. For example, specific EAA formulas might support growth of bifidobacterial subspecies important for infant gut barrier development and function [162], improved vaccine responses, such as the *Bifidobacterium longum* subsp. *Infantis* [163], or production of essential nutrients, including folate and riboflavin [164]. Completely undefined in infant development is the role of father’s microbiota and its changes, potentially induced by diet and dietary supplements.

Little is known about the influence of gender on gut microbiota composition, and how this factor can affect the efficacy of amino acid supplements [57, 120]. Few studies have been conducted to investigate the role that sex plays in development and age-related changes of microbiota composition, increasingly evident starting at puberty and most defined in adult and aged subjects [165]. It seems that males and females are uniquely susceptible to factors that shape the microbiota after birth. Male microbiota, in fact, provides testosterone-dependent protection from T1DM in a model of non-obese diabetic mice [166].

Several findings suggest bidirectional communication between the gut and the brain in behavioral, psychiatric, and neurodegenerative disorders. The microbiota regulates, in fact, expression of the 5-hydroxytryptamine receptor (5-HT_1A_), brain-derived neurotropic factor (BDNF), and NMDA receptor subunit 2 (NR2A) [167–169]. Thus, anxiety, hyperactivity, depression, nociception, and autism spectrum disorder are among the other psychiatric disorders to be linked to intestinal microbial communities [170–172]. Although the BCAAs do not act as direct precursors for neurotransmitters, they can affect transport of large neutral amino acids (LNAAs), including the BCAAs, across the blood–brain barrier, and thereby influence CNS concentrations of diverse neurotransmitters [173]. BCAAs can also be catalyzed in the astrocyte to produce glutamate and branched-chain α-keto acids, which are further taken up by neurons [174]. With the aim to reduce brain tyrosine uptake, BCAAs were given to bipolar subjects during periods of mania [175]. Sixty grams BCAAs were administered daily for 7 days and produced a significant reduction in manic symptoms, consistent with an effect on brain catecholamine. Gut microbiota might be hypothesized to play some role in this effect.

The gut microbes have recently been reported to promote α-synuclein pathology, neuroinflammation, and characteristic motor symptoms in a validated mouse model of Parkinson disease (PD). Notably, fecal microbes from PD patients impair motor function significantly more than microbiota from healthy controls when transplanted into mice [176]. Analogously, specific microbe ensembles influence stroke recovery in mice [177, 178], and amino acid supplements may potentiate this effect.

Although a body of knowledge is accumulating that suggests potential interactions between EAAs and gut microbiota and their effects on metabolic health and health span, the complex interplay between dietary amino acids and intestinal microbes remains largely unknown. In particular, it remains to be addressed whether the different effects of EAAs, acting either in catabolic or anabolic conditions, may be partially attributed also to differences in gut microbiota composition in these metabolic conditions. Furthermore, based on the current knowledge, the effects and metabolic fate of the dietary EAAs can be largely modified by different gut microbiota ensembles. Both EAA diet supplementation and gut microbiota contribute to human health acting at a systemic level. The precise interplay and the nature of their interactions are still poorly understood and they may help to predict more accurately the therapeutic effect of nutraceutical interventions with specific amino acid formulas.

### Conclusions and future perspectives

Studies of the human gut microbiota have changed how researchers view the pathophysiology of widely diffused metabolic disorders, particularly those linked to age. Humans co-evolved with a web of thousands of microbes, including not only bacteria, but also viruses, fungi and unicellular organisms called Archaea, with which strict relationship exists. Human intestine provides a comfortable environment and nutrients for microbes, and they digest food for us; in addition, they keep away pathogen microbes, synthesize vitamins, organize immune function, and transfer important messages to brain. Thus, it is possible that metabolic problems in humans could be managed with adequate care of the gut microbiota. Since the disturbance of microbial ecology and eco-systems are crucial for physiology in different human life periods, the knowledge of diet and dietary supplement impact on the gut microbiota might be very important for health. Dietary fibers and prebiotics—i.e., substances that induce the growth or activity of microorganisms contributing to the wellbeing of their host—are known to influence health in children and adults. We hypothesize that specific amino acid mixtures are likely to be of benefit to people who follow a typical Western-style diet, in addition to dietary fiber and prebiotics. A deeper understanding of the efficacy of such dietary supplements to maintain gut microbiota has the potential to contribute important therapeutic tools in human metabolic health and weight control.

## Abbreviations

5-HT_1A_: 5-Hydroxytryptamine receptor 1A
AKT: Serine-threonine protein kinase
AMPK: 5′ Adenosine monophosphate-activated protein kinase
BCAAem: BCAA-enriched mixture
BCAAs: Branched-chain amino acids
BDNF: Brain-derived growth factor
EAAs: Essential amino acids
GLP-1: Glucagon-like peptide 1
GPR41: G protein-coupled receptor 41
GPR43: G protein-coupled receptor 43
IL: Interleukin 10
LNAAs: Large neutral amino acids
mTOR: Mechanistic target of rapamycin
NR2A: N-methyl-D-aspartate receptor subunit 2
PD: Parkinson disease
PsA: Polysaccharide A
rRNA: Ribosomal ribonucleic acid
SCFAs: Short-chain fatty acids
T2DM: Type 2 diabetes mellitus
TMAO: Trimethylamine N-oxide
Treg: Regulatory T cell
